# Awake craniotomy and language assessment in deaf patients: a systematic review of feasibility, communication strategies, and outcomes

**DOI:** 10.1007/s11060-026-05560-0

**Published:** 2026-04-11

**Authors:** Mohammad Mofatteh, Mohammad Sadegh Mashayekhi, Keyoumars Ashkan

**Affiliations:** 1 School of Medicine, Dentistry and Biomedical Sciences, Queen’s University Belfast, Belfast, UK; 2 Department of Neurosurgery, Neuro International Collaboration (NIC), London, UK; 3 Division of Neurosurgery, Faculty of Medicine, University of Ottawa, Ottawa, ON Canada; 4 Department of Neurosurgery, Neuro International Collaboration (NIC), Vancouver, Ottawa, ON Canada; 5 School of Biomedical Engineering and Imaging Sciences, Faculty of Life Sciences and Medicine, King’s College London, London, UK; 6 King’s Health Partners Academic Health Sciences Centre, London, UK; 7 Department of Basic and Clinical Neuroscience, Institute of Psychiatry, Psychology and Neuroscience, King’s College London, London, UK; 8 Department of Neurosurgery, King’s College Hospital NHS Foundation Trust, London, UK

**Keywords:** Awake craniotomy, Awake brain surgery, Brain mapping, Hearing impairment, Speech, Deaf, Aphasia, Patient outcome, Inclusivity

## Abstract

**Introduction:**

Awake craniotomy (AC) relies on effective intraoperative communication for functional brain mapping and is often excluded in patients with hearing impairment. This exclusion is largely based on theoretical concerns rather than empirical evidence. This systematic review aimed to critically evaluate the feasibility, communication strategies, and reported neurological outcomes of AC in patients with hearing impairment.

**Methods:**

A systematic search of PubMed, Scopus, and Web of Science was conducted from inception to December 2025 in accordance with PRISMA guidelines. Only English-language studies reporting AC in patients with hearing impairment were included. Data were extracted on patient characteristics, communication strategies, intraoperative mapping paradigms, and postoperative neurological outcomes.

**Results:**

Eight single-center case reports published between 2017 and 2025 were included, comprising eight individual adult patients (5 males, 62.5%; mean age 55.57 ± 11.75 years, range 45–75). Seven patients (87.5%) used sign language-based intraoperative communication supported by professional interpreters, while one patient used a bone-conduction voice amplifier. Language mapping was reported in seven cases. No included report described intraoperative communication failure or conversion to general anesthesia; however, adverse event reporting was inconsistent across studies. Gross total or near-total resection was reported in three cases (37.5%). No permanent new postoperative neurological deficits were described, although follow-up duration and outcome reporting were heterogeneous.

**Conclusions:**

The available literature is limited to isolated case reports and provides very low-quality evidence. While these reports suggest that awake craniotomy can be technically feasible in highly selected hearing-impaired patients using individualized communication strategies, the findings are not generalizable and should be interpreted as hypothesis-generating. This review primarily highlights conceptual feasibility, identifies critical knowledge gaps, and proposes directions for future research rather than establishing safety or equivalence to standard AC populations.

**Clinical trial number:**

Not applicable.

**Supplementary Information:**

The online version contains supplementary material available at 10.1007/s11060-026-05560-0.

## Introduction

Awake craniotomy (AC) with intraoperative cortical and subcortical mapping has become an established standard of care for the resection of intrinsic and extrinsic brain lesions located within or adjacent to eloquent cortex [1–6]. By enabling real-time functional assessment during tumor resection, AC allows neurosurgeons to maximize the extent of resection while minimizing the risk of permanent postoperative neurological deficits [7–9]. Over the past two decades, refinements in anesthetic techniques, neurophysiological monitoring, and language-mapping paradigms have expanded the indications for AC, making it a cornerstone of contemporary functional neurosurgery [10–14].

Central to the success of AC is effective bidirectional communication between the patient and the surgical team. Intraoperative assessment of language, motor, cognitive, and visual functions depend on the patient’s ability to understand instructions and to reliably produce behavioral responses during direct electrical stimulation [15, 16]. Consequently, candidate selection for AC has traditionally emphasized intact comprehension and expressive capacity, and communication barriers, such as deafness, severe aphasia, or significant speech perception deficits, are frequently cited as relative or absolute contraindications in many centers [17–20]. While such exclusions are often pragmatic, they risk systematically denying selected patient populations access to the demonstrated oncological and functional benefits of awake mapping.

Hearing impairment represents one of the most common sensory disabilities worldwide, affecting hundreds of millions of individuals across diverse age groups [21]. It may arise congenitally or be acquired later in life as a consequence of aging, infection, trauma, or otologic disease. In neurosurgical practice, hearing impairment intersects with several challenges relevant to AC: reduced auditory perception, reliance on non-verbal communication strategies, and potential difficulties in expressing pain, discomfort, or neurological symptoms intraoperatively. These challenges are further compounded in patients who primarily use sign language, in whom linguistic expression is inherently motoric and visuospatial rather than auditory- verbal.

Sign languages are fully developed natural languages with distinct phonological, syntactic, and semantic structures. Unlike spoken language, which is produced orally and perceived auditorily, sign language is produced through coordinated hand movements, facial expressions, and body posture, and is perceived visually [22]. Neuroimaging and electrophysiological studies have demonstrated that sign language processing recruits classical perisylvian language networks, including the inferior frontal gyrus and posterior temporal regions, while also engaging visuomotor and parietal areas related to spatial processing and manual dexterity [23]. Importantly, cortical reorganization and cross-modal plasticity following auditory deprivation may further modify functional networks in deaf individuals, particularly in those with prelingual hearing loss [24, 25].

These neurobiological characteristics introduce unique complexities during AC. For example, stimulation-induced disruption of hand motor function may mimic or obscure language errors in signing patients, making it difficult to distinguish motor deficits from genuine linguistic impairment [26]. Similarly, the need to preserve bimanual dexterity during mapping imposes specific constraints on patient positioning, surgical exposure, and operating room setup [27]. Despite these challenges, emerging case reports suggest that, with appropriate preoperative planning and tailored intraoperative paradigms, often involving sign language interpreters or alternative communication technologies, AC appears to be technically feasible in carefully selected hearing-impaired patients when communication strategies are individualized and appropriately supported.

Nevertheless, the existing literature on AC in hearing-impaired patients remains sparse, fragmented, and largely limited to single-case reports or small case series. There is currently no consolidated synthesis of published evidence addressing the safety, feasibility, communication strategies, and neurological outcomes of AC in this population. This paucity of data perpetuates uncertainty among surgeons and anesthesiologists and may contribute to the continued exclusion of hearing-impaired patients from awake neurosurgical procedures, raising important ethical and equity considerations.

Given the persistent exclusion of hearing-impaired patients from awake neurosurgical protocols and the absence of consolidated evidence addressing this practice, a critical synthesis of the available literature is warranted. The aim of this systematic review was to summarize and appraise published case reports describing AC in patients with hearing impairment, with specific attention to intraoperative communication strategies, mapping paradigms, perioperative feasibility, and reported neurological outcomes. Importantly, this review does not seek to establish safety or efficacy, but rather to identify patterns, limitations, and knowledge gaps in the existing literature to inform future research and clinical decision-making.

## Materials and methods

### Inclusion and exclusion criteria

We employed a search strategy that was explained previously [16]. Articles were included if they met the following criteria: (1) original case reports or case series; (2) English-language publication; (3) human subjects; (4) AC performed in patients with documented hearing impairment, including congenital or acquired deafness; and (5) sufficient individual-level clinical detail to permit data extraction. Articles were excluded if they: (1) combined hearing-impaired and hearing patients without separable data; (2) involved exclusively general anesthesia; (3) lacked sufficient intraoperative or postoperative detail; or (4) were reviews, editorials, abstracts, or conference proceedings.

A prospective review protocol was not registered prior to study initiation. This review was therefore conducted as an exploratory synthesis of rare case reports, and findings should be interpreted accordingly. The absence of protocol registration reflects the limited and heterogeneous nature of the available literature and represents a methodological limitation of this study.

### Data source and search strategy

We conducted this systematic review following the Preferred Reporting Items for Systematic Reviews and Meta-Analysis (PRISMA) guidelines [28] to identify published literature on AC in patients with hearing impairment. A separate protocol was not established in this study. PubMed, Scopus, and Web of Science databases were searched from inception to December 23rd, 2025, for relevant articles. The following Boolean terms were used for the search: (“awake craniotomy” OR “awake brain surgery” OR “awake neurosurgery” OR “awake brain mapping” OR “awake tumor resection” OR “craniotomy while awake”). Given the rarity of AC in hearing-impaired patients, a deliberately broad search strategy was employed; however, this approach likely contributed to a high initial yield and may have reduced search specificity.

Details of search terms for each database are demonstrated in Supplementary Table 1.

### Article selection process

Initial screening of titles and abstracts was independently performed by two reviewers (M.M. and M.S.M.). Articles deemed potentially eligible underwent full-text review for final inclusion. Discrepancies were resolved by consensus. Reference lists of all included studies were additionally screened to identify relevant articles not captured by the database search.

### Data extraction and synthesis

Data extraction was independently performed by two authors (M.M. and M.S.M.) using a standardized data collection form and discrepancies resolved by consensus. Extracted variables included: first author, year of publication, article title, journal, country of study, study objectives, inclusion and exclusion criteria for AC, study period, study design, single- or multi-center status, pathology type, anesthesia protocol, primary sedative and analgesic agents, follow-up duration, total number of patients, comparison of AC with GA (if applicable), patient population (adult or pediatric), age, sex, handedness, operative duration, length of hospitalization, intraoperative complications, lesion hemisphere and lobe, eloquent-area involvement and mapping strategy, extent of resection, type of communication impairment, prelingual versus postlingual deafness, conversion to GA, preoperative neurological symptoms, postoperative complications and neurological deficits, and primary outcomes.

The methodological quality of included case reports was assessed using the Joanna Briggs Institute (JBI) Critical Appraisal Checklist for Case Reports [29]. This 8-item tool evaluates the clarity of patient demographics, history, clinical condition, diagnostics, intervention description, post-intervention condition, reporting of adverse events, and the presence of a takeaway lesson. Assessment was performed independently by two reviewers (M.M. and M.S.M.), with discrepancies resolved by consensus. Given the inherent limitations of the case report format, no study was excluded based on quality score; however, the results of this appraisal are synthesized to contextualize the strength of the evidence.

Given that all included studies were single-patient case reports, formal meta-analysis was not appropriate. Results are therefore presented as a descriptive qualitative synthesis, and numerical aggregation should not be interpreted as estimates of incidence, risk, or comparative effectiveness.

Extracted data were managed using Microsoft Excel (version 2016; Microsoft Corp., Redmond, WA, USA).

## Results

### Study selection

**Fig. 1:**
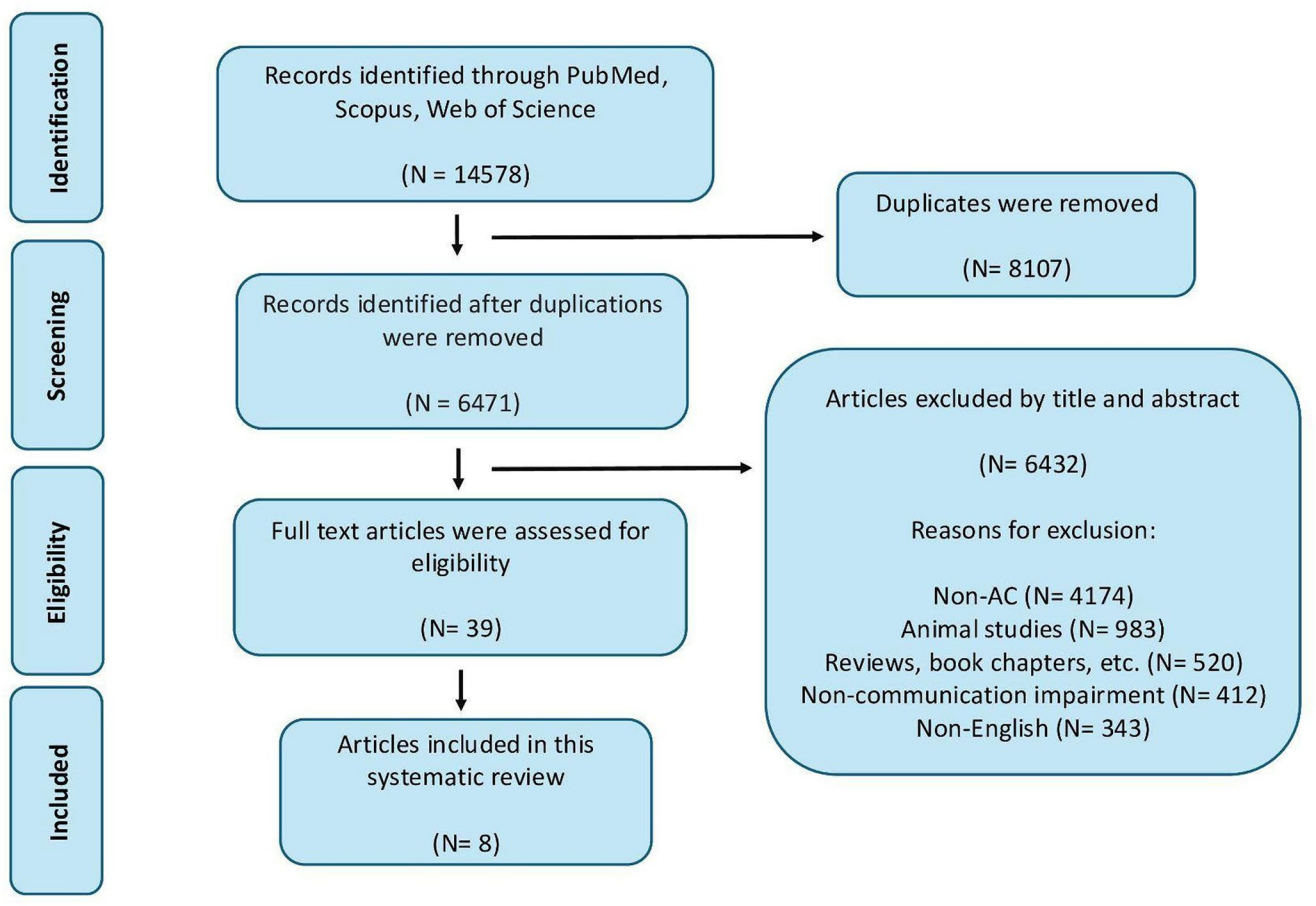
A flowchart of the current study demonstrating identification, screening, eligibility, and inclusion process according to the preferred reporting items for systematic reviews and meta-analyses (PRISMA). AC, awake craniotomy

The systematic search identified 14,578 records across PubMed, Scopus, and Web of Science. After removal of duplicates (*n* = 8,107; 55.6%), 6,471 records (44.4%) underwent title and abstract screening. Of these, 6,432 studies (99.4%) were excluded based on predefined criteria, leaving 39 full-text articles (0.27%) assessed for eligibility. Ultimately, eight studies were included in the qualitative synthesis. All were single-patient case reports, precluding any assessment of comparative outcomes or complication rates (Fig. 1).

A summary of included studies is shown in Table 1 [30–37].

**Table 1:** A summary of studies included

Study	Article title	Journal	Country	Objectives
Martino et al., 2017 [30]	Cross-Modal recruitment of auditory and orofacial areas during sign language in a deaf subject	World Neurosurgery	Spain	To describe an original case of a post-lingual deaf-mute patient undergoing AC with intraoperative electrical stimulation mapping for a left temporoinsular low-grade glioma.
Metellus et al., 2017 [31]	Successful insular glioma removal in a deaf signer patient during an awake craniotomy procedure	World Neurosurgery	France	To report a case of successful resection of a left insular glioma in a native deaf signer during an AC.
Tachibana et al., 2019 [32]	Successful awake craniotomy in an aged patient with severe hearing impairment using a bone conduction voice amplifier: a case report	JA Clinical Reports	Japan	To report a case of severe hearing impairment in an aged patient in whom AC was successfully performed with smooth communication by using a bone conduction voice amplifier.
Chen et al., 2020 [33]	Awake craniotomy for a left pan-hippocampal diffuse low-grade glioma in a deaf and mute patient using sign language	World Neurosurgery	Taiwan	To present a native deaf and mute patient who has been diagnosed with a left pan-hippocampal glioma undergoing AC using sign language during intraoperative monitoring.
Lau et al., 2023 [34]	Subcortical language localization using sign language and awake craniotomy for dominant posterior temporal glioma resection in a hearing-impaired patient	Acta Neurochirurgica	Canada	To report a case where both sign language and spoken language are tested in the same patient AC; to describe a pre-operative fMRI and baseline naming approach for language mapping; to discuss the neuroscientific implications of our awake mapping for hearing-impaired patients.
Yamamoto et al., 2024 [35]	Awake surgery for a deaf patient using sign language: A case report	Surgical Neurology International	Japan	To discuss a case of AC using sign language in a patient with hearing impairment with a low-grade glioma.
Ene et al., 2025 [36]	Language mapping during awake brain surgery in a deaf patient with a brain tumor: illustrative case	Journal of Neurosurgery	USA	To describe the case of a patient with congenital deafness, who has been sign language-dependent since birth, presenting with a diffuse right hemispheric brain glioma who underwent resection with an AC.
Silva et al., 2025 [37]	Awake craniotomy for a deaf patient: A case report	A&A Practice	USA	To present anesthetic technique, drug dosages, and environmental considerations for a representative deaf patient undergoing AC.

Abbreviations: AC, awake craniotomy; fMRI, functional magnetic resonance imaging

### Quality assessment of included studies

The JBI critical appraisal revealed significant methodological limitations consistent with the case report format (Table 2 and Supplementary Table 2). While patient demographics, intervention descriptions, and post-operative conditions were generally well-reported, critical weaknesses were universal: 1) No study provided a detailed, chronological patient history timeline; 2) Pre-operative baseline assessment of sign language proficiency was either absent or minimally described, limiting the objective interpretation of intraoperative language mapping; and 3) The reporting of adverse events or their explicit absence was unclear in five of eight studies, raising the possibility of selective outcome reporting bias. The overall very low quality of evidence necessitates caution in interpreting these findings.

**Table 2:**
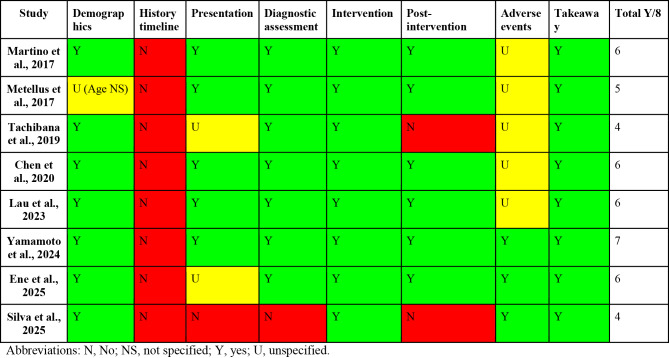
Detailed appraisal of each study using Joanna Briggs Institute (JBI) critical appraisal checklist

### Study characteristics

Study characteristics are summarized in Table 3. All included studies were single-center case reports (8/8, 100%), published between 2017 and 2025, reflecting the nascent and emerging nature of the literature on AC in patients with hearing impairment [30–37].

**Table 3:** A summary of studies characteristics

Study	Study period	Study design	Single/multi- center	Pathology	AC protocol	Surgical position	Primary sedation and analgesic	Operation length	Follow-up
Martino et al., 2017 [30]	2017	Case-report	Single-center	Glioma (low-grade)	Asleep-awake-asleep	Lateral	NS	NS	6 months
Metellus et al., 2017 [31]	2017	Case-report	Single-center	Astrocytic glioma (grade III)	NS	NS	NS	NS	NS
Tachibana et al., 2019 [32]	2019	Case-report	Single-center	Glioma	Asleep-awake-asleep	Lateral	Propofol, remifentanil, rocuronium, cranial nerve blocks, levobupivacaine	Awake phase: 123 min; total operation time: 484 min; anesthesia time: 621 min	NS
Chen et al., 2020 [33]	2020	Case-report	Single-center	Anaplastic ganglioglioma (grade III)	Asleep-awake-asleep	Supine	NS	NS	4 months
Lau et al., 2023 [34]	2023	Case-report	Single-center	Glioblastoma (IDH-wildtype, grade IV)	Asleep-awake-asleep	Supine	NS	NS	NS
Yamamoto et al., 2024 [35]	2024	Case-report	Single-center	Oligodendroglioma (grade II)	Asleep-awake-asleep	Lateral	NS	NS	1 year
Ene et al., 2025 [36]	2025	Case-report	Single-center	Diffuse infiltrative glioma	Awake-awake-awake	Side-lying position (lateral or semi-lateral position)	Liposomal bupivacaine with epinephrine	NS	15 months
Silva et al., 2025 [37]	2025	Case-report	Single-center	Glioma	Awake-awake-asleep	Lateral	Dexmedetomidine with remifentanil infusion, intermittent fentanyl and midazolam; scalp block with liposomal bupivacaine + epinephrine; dural lidocaine	12 hours	NS

Abbreviations: IDH, isocitrate dehydrogenase; NS, not specified

Reported pathologies were exclusively intrinsic brain tumors and encompassed a broad histopathological spectrum, including low-grade glioma [30], astrocytic glioma (grade III) [31], glioma (unspecified grade) [32, 37], anaplastic ganglioglioma (grade III) [33], glioblastoma (IDH-wildtype, grade IV) [34], oligodendroglioma (grade II) [35], and diffuse infiltrative glioma [36].

### Anesthetic technique and surgical setup

The asleep-awake–asleep anesthetic protocol was the most commonly employed approach, reported in five studies (5/8, 62.5%) [30, 32–35]. Patient positioning was most frequently lateral or semi-lateral, used in five studies (5/8, 62.5%) [30, 32, 35–37], while supine positioning was reported in two studies (2/8, 25.0%) [33, 34].

Operative duration was infrequently reported, with only two studies (2/8, 25.0%) providing detailed data on timing, documenting a total operative time of 484 minutes [32] and 12 hours [37]. Follow-up duration was reported in four studies (4/8, 50.0%), ranging from four months [33] to one year, which represented the longest reported follow-up period [35].

### Patient demographics and hearing impairment characteristics

A total of eight patients underwent AC across the included studies (Table 4). All patients were adults (7/8, 87.5% explicitly reported; 1/8, 12.5% age not specified) [31]. The mean age was 55.57 ± 11.75 years (range 45–75) in the seven studies reporting age (7/8, 87.5%) [30, 32–37].

**Table 4:** An overview of prevalence and demographics of patients from included studies

Study	Total patient number	Adults (≥18 years) or pediatrics (˂18 years)	Age (years) at the surgery	Sex	Communication impairment type	Prelingual/postlingual deafness	AC and GA comparison	Handedness
Martino et al., 2017 [30]	1	Adult	55	Female	Deaf and mute since the age 3 years after meningitis	Postlingual	No	Right-handed
Metellus et al., 2017 [31]	1	NS	NS	Male	Congenitally deaf, native user of sign language	Prelingual	No	Right-handed
Tachibana et al., 2019 [32]	1	Adult	75	Female	Severe hearing impairment due to advanced age and a history of myringoplasty	Postlingual	No	NS
Chen et al., 2020 [33]	1	Adult	58	Female	Deaf and mute	Postlingual	No	Right-handed
Lau et al., 2023 [34]	1	Adult	66	Male	No hearing on the right side and profound sensorineural hearing loss on the left side since childhood	Postlingual	No	Right-handed
Yamamoto et al., 2024 [35]	1	Adult	45	Male	Deaf but able to speak at a sufficiently audible level through childhood training	Prelingual	No	Right-handed
Ene et al., 2025 [36]	1	Adult	45	Male	Congenitally deaf	Prelingual	No	Right-handed
Silva et al., 2025 [37]	1	Adult	45	Male	Deafness requiring visual language communication	NS	No	NS

Abbreviations: AC, awake craniotomy; GA, general anesthesia; NS, not specified

Sex distribution was skewed toward males, with five male patients (5/8, 62.5%) and three female patients (3/8, 37.5%) among studies reporting sex [30–35, 37].

Regarding the timing of hearing impairment, four of the eight patients (50.0%) had postlingual deafness or severe hearing impairment, reported in the studies by Martino et al. [30], Tachibana et al. [32], Chen et al. [33], and Lau et al. [34]. The remaining three patients (37.5%) had prelingual or congenital deafness and were native users of sign language [31, 35, 36]. In one case (1/8, 12.5%), the timing of hearing impairment was not specified [37].

### Lesion characteristics and surgical indications

Lesions were predominantly located in the left hemisphere, accounting for 62.5% of reported cases (5/8), consistent with the predominance of dominant-hemisphere involvement in awake language-mapping surgery [30, 31, 33–35]. Right-hemispheric lesions were reported in three patients (3/8, 37.5%) [32, 36, 37] (Table 5).

**Table 5:** A summary of lesion characteristicses

Study	Lesion hemisphere	Lesion location	Lesion resection (Total/near total/subtotal, n, %)
Martino et al., 2017 [30]	Left (1, 100.0%)	Temporoinsular: 1 (100.0%)	NS
Metellus et al., 2017 [31]	Left (1, 100.0%)	Insular: 1 (100.0%)	Total (1, 100.0%)
Tachibana et al., 2019 [32]	Right (1, 100.0%)	Frontal: 1 (100.0%)	NS
Chen et al., 2020 [33]	Left (1, 100.0%)	Pan-hippocampal: 1 (100.0%)	Subtotal (1, 100.0%) [92.8% resection]
Lau et al., 2023 [34]	Left (1, 100.0%)	Temporal: 1 (100.0%)	Total (1, 100.0%)
Yamamoto et al., 2024 [35]	Left (1, 100.0%)	Frontal: 1 (100.0%)	Total (1, 100.0%)
Ene et al., 2025 [36]	Right (1, 100.0%)	Frontal and basal ganglial: 1 (100.0%)	Subtotal (1, 100.0%), [80% resection]
Silva et al., 2025 [37]	Right (1, 100.0%)	NS	NS

Abbreviations: NS, not specified

With respect to lobar distribution, lesions most frequently involved the frontal lobe (2/8, 25.0%) [32, 35]. Other locations included the temporal lobe (1/8, 12.5%) [34], the insular region (1/8, 12.5%) [31], the temporoinsular region (1/8, 12.5%) [30], pan-hippocampal involvement (1/8, 12.5%) [33], and combined frontal-basal ganglial involvement (1/8, 12.5%) [36]. One study (1/8, 12.5%) did not report lesion location [37].

### Extent of resection and intraoperative safety

Gross total or near-total resection was achieved in three patients (3/8, 37.5%) [31, 34, 35]. Subtotal resection (>90%) was reported in two patients (2/8, 25.0%) [33, 36]. The extent of resection was not specified in three studies (3/8, 37.5%) [30, 32, 37].

### Communication strategies and intraoperative mapping

Detailed operative characteristics were available for all included studies and are summarized in Table 6. Across the eight cases, eloquent-area mapping was performed in 87.5% of patients (7/8), with language mapping conducted in six patients (6/8, 75.0%) and combined language and motor mapping in four patients (4/8, 50.0%) [30, 31, 33, 36]. One patient (1/8, 12.5%) underwent isolated motor mapping due to preserved verbal comprehension facilitated by a bone conduction voice amplifier [32], while eloquent-area mapping was not specified in one study [37].

**Table 6:** A summary of operation details

Study	Eloquent area mapping	Eloquent area mapping method	Communication approach	Intraoperative complications	Conversion to GA (n)	Hospitalization length
Martino et al., 2017 [30]	Sensorimotor, language	Counting, picture naming, text reading, N-back memory task	Sign language	NS	None	NS
Metellus et al., 2017 [31]	Language and motor	Picture naming, text reading, repetition, perception	Sign language	None	None	NS
Tachibana et al., 2019 [32]	Motor	Following instructions, hand movement	Bone conduction voice amplifier	None	None	NS
Chen et al., 2020 [33]	Language and motor	Number counting, visual object naming, continuous motor function	Sign language	None	None	NS
Lau et al., 2023 [34]	Language	Neuromapper	American Sign Language	None	None	NS
Yamamoto et al., 2024 [35]	Language	Counting, picture naming, reading	Sign language	None	None	9 days
Ene et al., 2025 [36]	Language and motor	Number counting, visual object naming, continuous motor function	American and British Sign Languages	None	None	3 days
Silva et al., 2025 [37]	Language	NS	American Sign Language	None	None	NS

Abbreviations: GA, general anesthesia; NS, not specified

Sign language was the dominant intraoperative communication strategy, employed in seven cases (7/8, 87.5%) [30, 31, 33–37]. In these cases, communication relied on real-time visual interaction between the patient, a professional sign language interpreter, and the neuropsychology team. Both American Sign Language and British Sign Language were used, depending on patient background [34, 36].

Only one study (1/8, 12.5%) used a non-sign-based strategy, using a bone conduction voice amplifier to allow reliable verbal instruction-following in a patient with severe age-related hearing impairment [32]. This approach enabled effective execution of motor tasks without requiring manual communication.

Intraoperative tasks included number counting (3/8, 37.5%) [30, 33, 36], visual object or picture naming (5/8, 62.5%) [30, 31, 33–35], text reading (3/8, 37.5%) [30, 31, 35], repetition (2/8, 25.0%) [31, 34], continuous motor testing (3/8, 37.5%) [30, 33, 36], and working memory tasks (1/8, 12.5%) [30]. Intraoperative task was not specified in one study [37].

### Intraoperative safety and tolerance

None of the included case reports explicitly described intraoperative abandonment of mapping or conversion to general anesthesia due to communication failure. However, reporting of intraoperative adverse events was inconsistent, and the absence of reported complications cannot be interpreted as evidence of safety. All patients were able to comply with intraoperative tasks throughout the awake phase. No patient required conversion from AC to GA (0/8, 0.0%), underscoring the feasibility of these tailored communication strategies.

Seizure activity was reported in isolated cases but did not necessitate abandonment of mapping or termination of the awake procedure [31, 34]. These events were managed successfully using standard intraoperative protocols.

Length of hospitalization was reported in two studies (2/8, 25.0%), with a postoperative stay of 9 days in one patient [35] and 3 days in another [36]. No prolonged hospitalization attributable to awake technique or communication barriers was reported.

### Preoperative presentation and postoperative neurological outcomes

Major operative outcomes, including preoperative neurological status, postoperative deficits, and overall surgical results, are summarized in Table 7. Preoperative neurological symptoms were reported in six of eight patients (6/8, 75.0%), most commonly seizures (3/8, 37.5%) [30, 34, 36]. Other presenting symptoms included focal sensory disturbances (1/8, 12.5%) [31], vertigo (1/8, 12.5%) [33], and headache (1/8, 12.5%) [35]. Preoperative language-related disturbances affecting sign production, such as formational errors and impaired hand-shape sequencing, were described in one patient (1/8, 12.5%) with a dominant insular lesion [31].

**Table 7:** Major operative outcomes

Study	Preoperative neurological deficits and presentation	Postoperative neurological deficits	Main outcome
Martino et al., 2017 [30]	Epileptic seizure	Transient mild dysphasia (resolved within 2 weeks)	Sign-language–based intraoperative mapping during AC was feasible and comparable to oral-language mapping. Integration of a sign language interpreter with the neuropsychology team was essential. Cortical stimulation demonstrated cross-modal recruitment of auditory and orofacial motor regions during signed language processing.
Metellus et al., 2017 [31]	Right facial and upper-limb paresthesia; preoperative sign-language production errors (hand-shape and sequencing difficulties); focal seizures with secondary generalization	None	AC with direct electrical stimulation enabled the complete resection of a left insular tumor in a native deaf signer without postoperative neurological deficits, confirming the feasibility of language mapping in sign language users.
Tachibana et al., 2019 [32]	NS	NS	AC was safely performed in an elderly patient with severe hearing impairment using a bone conduction voice amplifier, demonstrating feasibility of non–sign-based communication strategies under careful anesthetic management.
Chen et al., 2020 [33]	Vertigo during squatting	None	Sign language-based intraoperative monitoring allowed safe awake resection of a pan-hippocampal glioma, achieving > 90% extent of resection in a single-stage procedure without postoperative neurological deficits.
Lau et al., 2023 [34]	Focal impaired awareness seizure	NS	AC with cortical and subcortical mapping was feasible in a hearing-impaired patient using both signed and spoken languages, enabling effective assessment of receptive and expressive language pathways.
Yamamoto et al., 2024 [35]	Mild headache	None	AC with tailored intraoperative tasks based on the degree of hearing impairment enabled safe tumor resection without postoperative neurological deterioration.
Ene et al., 2025 [36]	Seizure	Mild motor problems	Continuous cortical stimulation combined with sign-language–based intraoperative tasks enabled safe subtotal resection of a right hemispheric glioma in a congenitally deaf patient, preserving signed language function and highlighting the involvement of classical language regions, including Exner’s area, in signed language production in signed language production.
Silva et al., 2025	NS	NS	AC is successful with preserved communication and language function in a deaf patient. The patient tolerated procedure well with no intraoperative complications

Abbreviations: AC, awake craniotomy; NS, not specified

Overall, no permanent new postoperative neurological deficits were reported in any patient (0/8, 0.0%). Transient postoperative neurological changes occurred in two patients (2/8, 25.0%). One patient (1/8, 12.5%) developed mild dysphasia following left temporoinsular tumor resection, which resolved completely within two weeks [30]. Another patient (1/8, 12.5%) experienced mild postoperative motor impairment following subtotal resection of a right hemispheric diffuse glioma; this deficit was not reported as permanent or functionally disabling [36].

No postoperative worsening of signed language function was reported in any patient undergoing sign language–based intraoperative mapping (6/8, 75%) [30, 31, 33–36]. In these cases, patients preserved the ability to produce fluent and grammatically correct signed language postoperatively, including those with dominant-hemisphere and insular lesions. One study did not specify preoperative and postoperative complications [37].

### Seizure control and functional outcomes

In patients presenting with seizures preoperatively (3/8, 37.5%), postoperative seizure control was reported as favorable in all cases, with no new seizure burden attributed to the awake procedure [30, 34, 36].

Functional outcomes were uniformly reported as favorable across studies. All authors concluded that AC enabled safe tumor resection while preserving neurological function and communication ability. In particular, studies emphasized that sign-language–based intraoperative mapping allowed reliable identification of critical language cortex and subcortical pathways, even in patients with congenital deafness [31, 36].

## Discussion

AC with intraoperative cortical and subcortical mapping is widely regarded as standard-of-care approach for resection of lesions involving eloquent brain regions, allowing maximal extent of resection while minimizing permanent neurological morbidity. Large series and meta-analyses have consistently demonstrated the safety and functional superiority of AC compared with surgery under general anesthesia, particularly for dominant-hemisphere tumors and low-grade gliomas. However, despite these advances, patient selection criteria in many centers continue to exclude individuals with communication impairments, most notably hearing loss, largely on theoretical rather than evidence-based grounds.

Case reports included in this systematic review suggest that AC appears to be technically feasible in carefully selected hearing-impaired patients when communication strategies are individualized and appropriately supported. Across all included cases, there were no permanent postoperative neurological deficits, no conversions to general anesthesia, and there was preservation of communication abilities, including signed language. These outcomes appear qualitatively similar to those reported in large AC series; however, meaningful comparison is not possible given the absence of controlled data.

The absence of reported permanent neurological deficits or conversion to general anesthesia across published cases should not be misconstrued as evidence of procedural safety. Negative outcomes, aborted procedures, or unsuccessful communication attempts are less likely to be reported, particularly in rare and technically demanding scenarios. Consequently, the findings of this review should not be extrapolated to broader clinical practice without reservation.

### Awake craniotomy and communication barriers: reassessing traditional contraindications

Effective bidirectional communication is central to AC, particularly for language and cognitive mapping. Consequently, impaired comprehension or expressive capacity has traditionally been viewed as a relative or absolute contraindication. This stance mirrors earlier reservations regarding AC in elderly patients, individuals with mild aphasia, or those with psychiatric comorbidities [38–40], groups that were later shown to tolerate awake procedures safely when carefully selected and supported.

The present findings align with a growing body of literature challenging rigid exclusion criteria for AC. Studies examining AC in elderly populations, patients with preoperative language deficits, and even pediatric cohorts have demonstrated that awake mapping remains feasible when neuropsychological paradigms are adapted to the patient’s baseline abilities [41]. Hearing impairment should be viewed similarly: not as a categorical exclusion, but as a variable requiring tailored intraoperative assessment.

### Language organization beyond audition: insights from sign language and neuroplasticity

One of the most important contributions of the studies reviewed lies in their demonstration that signed language can be reliably mapped intraoperatively using direct electrical stimulation. Neuroimaging studies over the past two decades have shown that sign languages recruit classical left-hemisphere language regions- including the inferior frontal gyrus, posterior superior temporal gyrus, and supramarginal gyrus- despite being produced manually and perceived visually [42–45]. While stimulation-induced disruptions of signed language reported in several cases are consistent with existing neuroimaging literature, these observations remain anecdotal and cannot independently validate modality-independent models of language organization. Rather, they provide illustrative examples that support the biological plausibility of sign-language mapping during awake surgery.

The intraoperative findings reported by studies reviewed here provide rare causal evidence supporting these models. Stimulation-induced errors affecting sign formation, lexical retrieval, and semantic processing occurred in regions analogous to those responsible for spoken language disruption in hearing competent patients. Importantly, these errors were distinguishable from pure motor deficits, emphasizing the need for experienced interpreters and baseline linguistic assessment.

Differences between prelingual and postlingual deafness further enrich this discussion. Neuroplastic reorganization following early auditory deprivation has been shown to enhance visuospatial and sensorimotor integration, with increased recruitment of parietal and occipital cortices [46, 47]. Despite these adaptations, the preservation of core perisylvian language networks observed intraoperatively suggests that fundamental language architecture remains conserved, reinforcing the rationale for applying standard language-preservation principles during AC in deaf patients.

### Surgical outcomes in the context of the awake craniotomy literature

The absence of permanent neurological morbidity in this cohort is particularly noteworthy given that most lesions involved the dominant hemisphere and eloquent cortex. In large AC series, permanent language deficits are reported in approximately 1–5% of patients, with transient deficits occurring more frequently [7, 40, 48, 49]. The outcomes observed here fall well within, and arguably below, these expected ranges, although firm conclusions are limited by sample size.

The extent of resection achieved in hearing-impaired patients was comparable to that reported in conventional AC cohorts, suggesting that awake approaches can be technically implemented without clear compromise to resection goals in reported cases. Importantly, no study reported communication failure as a limiting factor for resection, and no patient required conversion to general anesthesia, an outcome often cited as a surrogate marker of intraoperative feasibility.

Although no study directly compared anesthetic strategies in hearing-impaired versus hearing patients, several practical considerations emerge. Maintenance of patient alertness and visual engagement is particularly critical, as communication is predominantly visual rather than auditory. Excessive sedation may therefore disproportionately impair interaction compared to standard awake craniotomy. Furthermore, clear visual access to the interpreter must be preserved throughout anesthetic transitions. Future studies should explore whether specific anesthetic regimens, including dexmedetomidine-based protocols, offer advantages in optimizing cooperation and communication in this population.

### Towards a practical clinical framework

Beyond establishing feasibility, this review highlights the need for a standardized, practical framework for AC in deaf and hearing-impaired patients. Key operational considerations include:

1. Interpreter integration: The presence of a qualified medical sign language interpreter is non-negotiable for Deaf patients. Protocols must define interpreter positioning (maintaining patient sightlines while preserving sterility), role clarity (as a communication conduit rather than a clinical decision-maker), and structured preoperative briefing with the surgical, anesthetic, and neuropsychology teams. Importantly, for centers without local access to sign language expertise, remote interpretation via secure real-time video link represents a pragmatic alternative [50]. Cross-institutional tele-interpretation may allow experienced sign language specialists to support AC procedures remotely, ensuring equitable access to care while maintaining communication fidelity.
2. Operating room configuration: The patient’s head position, surgical draping, and equipment placement must preserve an unobstructed visual field between the patient, interpreter (in-person or on-screen), and the neurophysiological evaluating team. When remote interpretation is used, monitor placement should be optimized to maintain continuous visual engagement without interfering with surgical workflow or sterility.
3. Task adaptation: Language mapping paradigms must be validated for sign language, assessing phonology (handshape, movement, location), syntax, and semantics rather than auditory repetition or oral reading. Baseline preoperative assessment by an intra-operative assessor familiar with sign linguistics is essential. In addition, emerging technologies such as virtual reality (VR) and augmented reality (AR) offer promising tools for standardized task delivery [51]. VR/AR platforms or pre-recorded videos of expert signers can guide patients through language and motor tasks intraoperatively, particularly in settings lacking on-site sign language specialists. These technologies may also enhance reproducibility and reduce inter-operator variability, building on prior work demonstrating the feasibility of AR/VR-based task presentation in awake neurosurgical mapping. For motor mapping, VR and/or AR systems can be used to prompt visually mediated motor tasks using sign language or gesture-based instructions. Such approaches may improve task clarity, patient engagement, and standardization, particularly when combined with recorded or remotely delivered instructions from experienced sign language experts.
4. Pain, anxiety, and discomfort assessment also warrant special attention, as hearing-impaired patients may be less able to spontaneously express distress [16]. Proactive, visually mediated communication and close collaboration between anesthesiology, neuropsychology, and interpreting staff are therefore essential to ensure patient safety and comfort.

These requirements represent significant but surmountable logistical adaptations, analogous to those already made for intraoperative visual field or cognitive testing.

In addition to adapting conventional tasks, there is a clear need to develop and standardize sign language-specific intraoperative paradigms that probe distinct linguistic domains independent of auditory processing. Potential approaches include:

(i) Semantic association tasks, such as picture–picture matching or the Pyramid and Palm Trees Test (PPTT) [52], allowing assessment of conceptual processing without reliance on speech;
(ii) Syntax-based tasks using written sentence completion paradigms, in which patients select or produce grammatically correct signed responses based on visual prompts;
(iii) Lexical retrieval tasks adapted to sign language, including object-to-sign mapping or verb generation based on visual stimuli;
(iv) Multimodal matching paradigms, such as those described in GENTasks (https://www.gentasks.eu), where multiple objects are presented and patients are required to match them with written or conceptual cues.

These approaches may allow more granular assessment of phonological (handshape, movement, location), semantic, and syntactic processing in sign language users, thereby improving the precision and reproducibility of intraoperative language mapping in this population.

### Proposed step-by-step clinical workflow

Based on synthesis of available reports and established awake craniotomy principles, we propose a practical stepwise approach for managing hearing-impaired patients:

Preoperative phase:

1. Multidisciplinary assessment including neurosurgery, anesthesiology, neuropsychology, and a certified sign language interpreter.
2. Baseline evaluation of communication modality (sign language proficiency, lip reading, written comprehension).
3. Preoperative rehearsal of intraoperative tasks with the interpreter and neuropsychology team.
4. Planning of operating room layout to ensure uninterrupted visual communication.

Intraoperative phase:

1. Positioning to maintain direct line-of-sight between patient and interpreter (in-person or screen-based).
2. Continuous visual communication with standardized, pre-rehearsed commands.
3. Use of adapted task paradigms targeting semantic, syntactic, and motor-language domains.
4. Real-time monitoring of patient comfort using visually mediated feedback systems.

Postoperative phase:

1. Early reassessment of signed language function using the same preoperative paradigms.
2. Documentation of any transient or permanent deficits using standardized descriptors.
3. Integration of findings into institutional protocols to improve reproducibility.

This structured approach may help reduce variability, improve intraoperative reliability, and support broader implementation of awake craniotomy in this population.

### Ethical and equity considerations

Beyond technical feasibility, the findings of this review raise important ethical considerations. Denying AC to patients solely on the basis of hearing impairment risks perpetuating inequities in access to function-preserving neurosurgical care. As AC is increasingly recognized as a standard-of-care intervention for eloquent-region tumors, exclusion based on sensory disability-without evidence of increased risk- becomes difficult to justify.

The broader neurosurgical literature increasingly emphasizes inclusivity, shared decision-making, and individualized risk assessment [53]. Incorporating hearing-impaired patients into awake mapping protocols aligns with these principles and reflects a maturation of functional neurosurgery toward truly patient-centered practice.

## Limitations

The major limitation of this systematic review is the low quality and high risk of bias in the primary evidence base, as confirmed by our formal critical appraisal. The included studies are exclusively single-case reports, a design incapable of establishing safety or efficacy. Key methodological flaws include: (i) the near-universal lack of standardized pre-operative assessment for sign language users, making intraoperative ‘language’ mapping subjective and poorly quantifiable; (ii) inconsistent and likely selective reporting of adverse events, indicating a high probability of publication bias where only successful cases are documented; and (iii) the absence of comparative groups or long-term, standardized outcome measures. Consequently, the aggregated results represent a collection of expert, anecdotal successes rather than generalizable evidence. The reported 0.0% conversion to GA and 0.0% permanent deficit rate almost certainly reflects this bias and should not be interpreted as the true complication rate in broader clinical practice. Reporting was heterogeneous, with incomplete data on operative duration, follow-up, and neuropsychological outcomes in several studies. Publication bias is also likely, as unsuccessful or aborted cases may be underreported. Consequently, these findings should be interpreted cautiously and viewed as hypothesis-generating rather than definitive. Future multicenter registries or prospective observational studies are needed to better define best practices, standardize intraoperative protocols, and assess long-term functional and oncological outcomes in this population. Importantly, the absence of reported communication failure or major complications across included studies should be interpreted with caution, as unsuccessful or aborted procedures are unlikely to be published.

Importantly, this work should be viewed as an initial step toward redefining eligibility criteria for AC, shifting from exclusion based on sensory impairment toward individualized functional assessment.

## Conclusion

In appropriately selected patients, hearing impairment should not be considered an absolute contraindication to AC. The available evidence demonstrates that AC appears to be technically feasible in carefully selected cases in hearing-impaired patients using tailored communication strategies, including sign language and alternative auditory technologies. Preservation of language and neurological function is achievable, even in dominant-hemisphere and eloquent-region surgery. Broader adoption of inclusive awake mapping protocols may expand access to function-preserving neurosurgical care for patients with sensory communication impairments.

## Electronic supplementary material

Below is the link to the electronic supplementary material.

Supplementary Material 1

## Data Availability

No datasets were generated or analysed during the current study.
