# Supplementary material for: Awake craniotomy and language assessment in deaf patients: a systematic review of feasibility, communication strategies, and outcomes
Source: J Neurooncol. 2026 Apr 11;177(2):97. doi: 10.1007/s11060-026-05560-0 (PMC13070071; doi:10.1007/s11060-026-05560-0)
Supplement: Supplementary file 1 — Supplementary Material 1 [file 11060_2026_5560_MOESM1_ESM.docx]

**Supplementary Table 1.** An Overview of Search Terms Used for Each Database

| **Database** | **Search Results** | **Search Terms** |
| --- | --- | --- |
| **PubMed** | 4663 | (("awake"[All Fields] OR "awakeness"[All Fields] OR "awakes"[All Fields] OR "awaking"[All Fields]) AND ("craniotomy"[MeSH Terms] OR "craniotomy"[All Fields] OR "craniotomies"[All Fields])) OR (("awake"[All Fields] OR "awakeness"[All Fields] OR "awakes"[All Fields] OR "awaking"[All Fields]) AND ("brain"[MeSH Terms] OR "brain"[All Fields] OR "brains"[All Fields] OR "brain s"[All Fields]) AND ("surgery"[MeSH Subheading] OR "surgery"[All Fields] OR "surgical procedures, operative"[MeSH Terms] OR ("surgical"[All Fields] AND "procedures"[All Fields] AND "operative"[All Fields]) OR "operative surgical procedures"[All Fields] OR "general surgery"[MeSH Terms] OR ("general"[All Fields] AND "surgery"[All Fields]) OR "general surgery"[All Fields] OR "surgery s"[All Fields] OR "surgerys"[All Fields] OR "surgeries"[All Fields])) OR (("awake"[All Fields] OR "awakeness"[All Fields] OR "awakes"[All Fields] OR "awaking"[All Fields]) AND ("neurosurgery"[MeSH Terms] OR "neurosurgery"[All Fields] OR "neurosurgeries"[All Fields] OR "neurosurgery s"[All Fields] OR "neurosurgical procedures"[MeSH Terms] OR ("neurosurgical"[All Fields] AND "procedures"[All Fields]) OR "neurosurgical procedures"[All Fields])) OR (("awake"[All Fields] OR "awakeness"[All Fields] OR "awakes"[All Fields] OR "awaking"[All Fields]) AND ("brain mapping"[MeSH Terms] OR ("brain"[All Fields] AND "mapping"[All Fields]) OR "brain mapping"[All Fields]) AND ("awake"[All Fields] OR "awakeness"[All Fields] OR "awakes"[All Fields] OR "awaking"[All Fields]) AND ("cysts"[MeSH Terms] OR "cysts"[All Fields] OR "cyst"[All Fields] OR "neurofibroma"[MeSH Terms] OR "neurofibroma"[All Fields] OR "neurofibromas"[All Fields] OR "tumor s"[All Fields] OR "tumoral"[All Fields] OR "tumorous"[All Fields] OR "tumour"[All Fields] OR "neoplasms"[MeSH Terms] OR "neoplasms"[All Fields] OR "tumor"[All Fields] OR "tumour s"[All Fields] OR "tumoural"[All Fields] OR "tumourous"[All Fields] OR "tumours"[All Fields] OR "tumors"[All Fields]) AND ("resect"[All Fields] OR "resectability"[All Fields] OR "resectable"[All Fields] OR "resectates"[All Fields] OR "resected"[All Fields] OR "resecting"[All Fields] OR "resection"[All Fields] OR "resectional"[All Fields] OR "resectioned"[All Fields] OR "resectioning"[All Fields] OR "resections"[All Fields] OR "resective"[All Fields] OR "resects"[All Fields])) OR (("craniotomy"[MeSH Terms] OR "craniotomy"[All Fields] OR "craniotomies"[All Fields]) AND ("awake"[All Fields] OR "awakeness"[All Fields] OR "awakes"[All Fields] OR "awaking"[All Fields])) |
| **Scopus** | 5701 | ALL ( awake AND craniotomy OR awake AND brain AND surgery OR awake AND neurosurgery OR awake AND brain AND mapping AND awake AND tumour AND resection OR craniotomy AND while AND awake ) |
| **Web of Science** | 4214 | ALL=(awake craniotomy OR awake brain surgery OR awake neurosurgery OR awake brain mapping OR awake tumour resection OR craniotomy while awake) |

**Supplementary Table 2.** Domain-by-Domain Analysis (Aggregated Results) of Each Study Using Joanna Briggs Institute (JBI) Critical Appraisal Checklist for Case Reports (2020 version)

| **JBI Item** | **Key Criterion** | **Aggregate Compliance (n=8)** | **Major Issues Identified** |
| --- | --- | --- | --- |
| **1** | Were the patient's demographic characteristics clearly described? | High (7/8 Yes) | One study (Metellus 2017) did not specify patient age. All others described age and sex. |
| **2** | Was the patient’s history clearly described and presented as a timeline? | Low (0/8 Yes) | Major Weakness. Most reports mention pre-op symptoms (e.g., seizures) but lack a detailed, chronological history from symptom onset to diagnosis to pre-op management. |
| **3** | Was the current clinical condition of the patient on presentation clearly described? | Moderate (5/8 Yes) | Most described presenting symptoms (seizure, headache). Two were less specific (Tachibana 2019: "severe hearing impairment"; Ene 2025: presentation details sparse). |
| **4** | Were diagnostic tests or assessment methods and the results clearly described? | Moderate (7/8 Yes) | MRI findings were described. However, detailed pre-operative neuropsychological assessment of sign language proficiency (e.g., formal assessment of phonology, syntax, semantics in sign language) was universally absent or minimally described, a major gap for language mapping studies. |
| **5** | Was the intervention(s) or treatment procedure clearly described? | High (8/8 Yes) | The AC procedure, anesthetic protocol (where specified), and use of interpreter/device were clearly described in all reports. |
| **6** | Was the post-intervention clinical condition clearly described? | High (6/8 Yes) | Immediate post-op neurological status was reported in most. One study (Tachibana 2019) lacked specific post-op neurological details. |
| **7** | Were adverse events (harms) or unanticipated events identified and described? | Moderate (3/8 Yes) | Critical Weakness. Only 4 studies explicitly stated "no complications" or described events (e.g., seizures). The others did not explicitly address adverse events, leaving it unclear if none occurred or if they were unreported. This raises serious concerns about selective outcome reporting bias. |
| **8** | Does the case report provide takeaway lessons? | High (8/8 Yes) | All studies concluded that AC was feasible, thus providing a clinical "lesson." |

Abbreviations: AC, awake craniotomy
